# Successful endoscopic submucosal dissection of a large laterally spreading tumor in distal jejunum by the water pressure method using a newly developed double-balloon enteroscope

**DOI:** 10.1055/a-2751-0170

**Published:** 2026-01-08

**Authors:** Shoma Murata, Kaoru Takabayashi, Motoki Sasaki, Kumiko Kirita, Motohiko Kato

**Affiliations:** 1 592818 Division of Gastroenterology and Hepatology, Department of Internal Medicine, Keio University School of Medicine, Tokyo, Japan; 2 592818 Center for Diagnostic and Therapeutic Endoscopy, Keio University School of Medicine, Tokyo, Japan; 3 592818 Division of Research and Development for Minimally Invasive Treatment, Cancer Center, Keio University School of Medicine, Tokyo, Japan

Despite advances in therapeutic endoscopy, reports of endoscopic resection in the small intestine remain extremely limited, and only a few cases of endoscopic submucosal dissection (ESD) for proximal small intestinal lesions without deep intubation have been reported 1 . Here, we present a case of distal jejunal ESD performed using a newly developed double-balloon enteroscope ( Video 1 ).

Successful endoscopic submucosal dissection of a large laterally spreading tumor in the distal jejunum by the water pressure method using a newly developed double-balloon enteroscope.Video 1

**Fig. 1:**
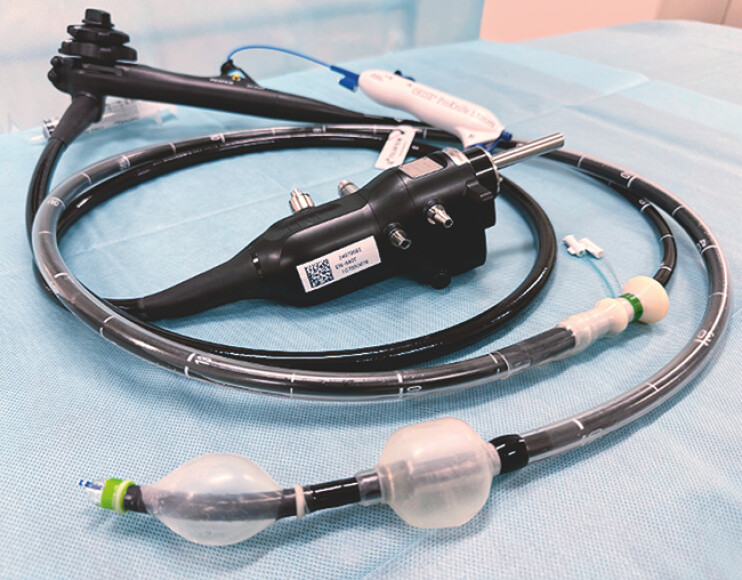
Appearance of the double-balloon enteroscope with the energy device in place. The energy device used for jejunal ESD was the ORISE 1.5-mm ProKnife (Boston Scientific Japan, Tokyo, Japan). ESD, endoscopic submucosal dissection.

**Fig. 2:**
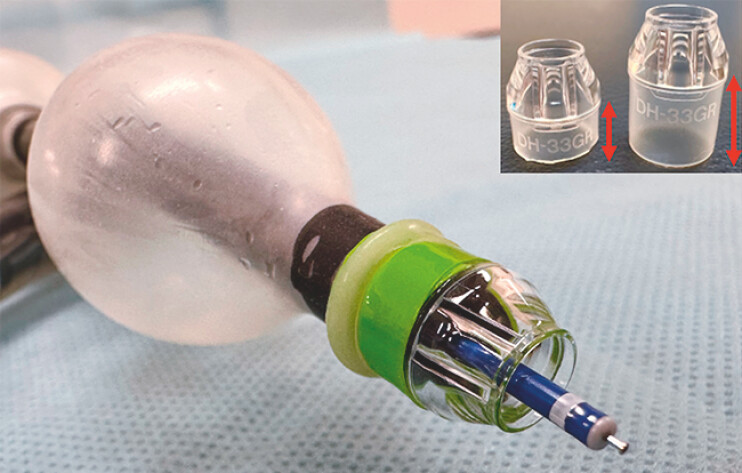
The tip of the double-balloon enteroscope. The ST-hood was modified by cutting off the rubber part and then attached to the tip of the enteroscope, and the balloon tip of the enteroscope also functioned without issues.

**Fig. 3:**
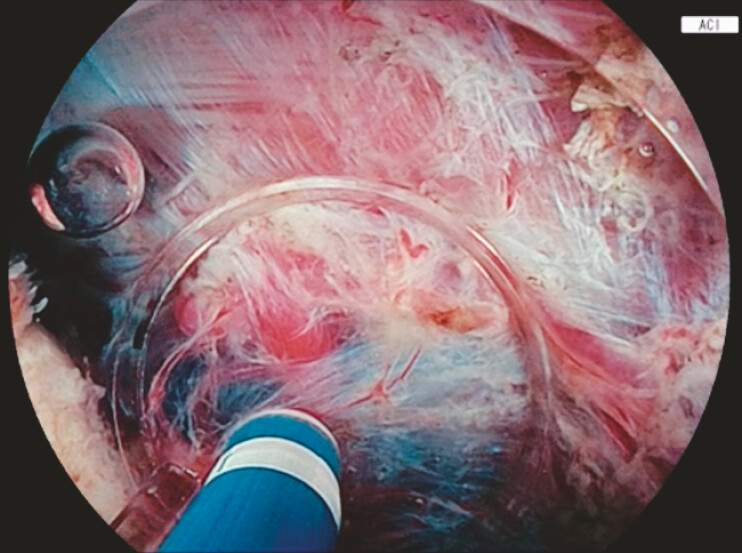
Amber-red color imaging (ACI) during the ESD with the water pressure method. ACI enhances the visualization of the dissection plane within the very thin submucosal layer.

**Fig. 4:**
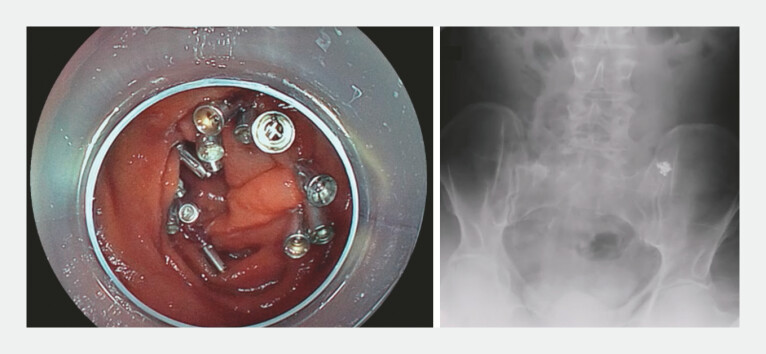
A white light image and an abdominal radiography after complete suturing of the mucosal defect. The mucosal defect was completely closed by the origami method using endoscopic clips (SureClip; Boston Scientific Japan, Tokyo, Japan). The radiographic image indicates that the treated lesion was identified in the pelvis, and the procedure was completed without perforation.

**Fig. 5:**
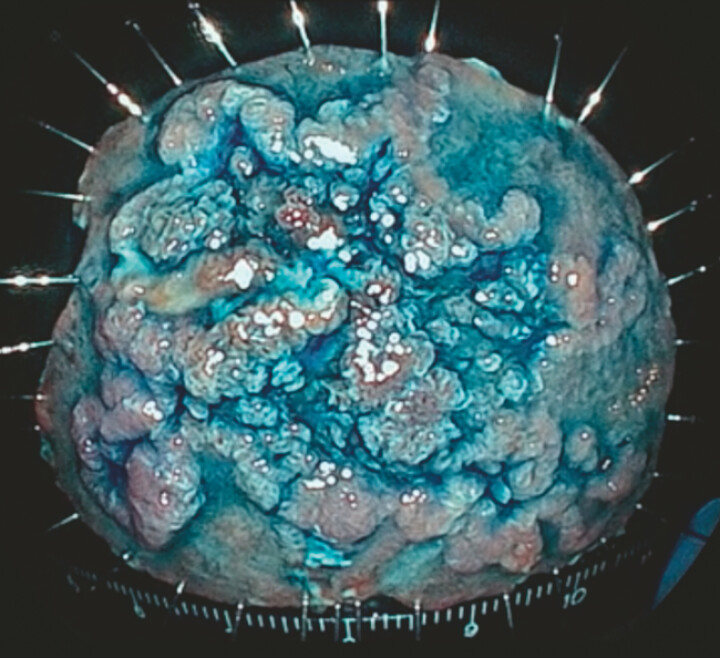
A white light image of the specimen with indigo carmine. En-bloc resection was successfully achieved. Pathological diagnosis: Tubular adenoma, intestinal type, pHM0, and pVM0.

A man in his fifties with familial adenomatous polyposis was found to have a large tumor in the small intestine. He had a history of total colectomy for rectal cancer. Because of the lesion size, we performed ESD using a double-balloon enteroscope (EN-840T, Fujifilm, Tokyo, Japan). The enteroscope was inserted trans-orally and advanced to the distal jejunum in the pelvis, where a 50-mm laterally spreading tumor was identified. Submucosal injection of hyaluronic acid was initiated from the distal side, followed by complete circumferential mucosal incision. Submucosal dissection was then attempted; however, it was extremely difficult due to poor maneuverability from prior surgery and the narrow submucosal space. Therefore, the enteroscope was withdrawn, and a small-caliber transparent hood (ST hood: DH-33GR, Fujifilm) was attached ( Fig. 1 , Fig. 2 ). The water jet function was then applied for the water pressure method (WPM), which facilitated entry into the narrow submucosal space ( Fig. 3 ) 2 3 . Repeated balloon assisted re-positioning also stabilized maneuverability. Finally, en bloc resection was successfully achieved in 263 minutes without perforation, and the mucosal defect was completely closed with clips ( Fig. 4 , Fig. 5 ). The postoperative course was uneventful except for mild pancreatitis, which resolved conservatively, and the patient was discharged on postoperative day 6.

To the best of our knowledge, this is the first successful case of distal jejunal ESD. The newly developed double-balloon enteroscope enabled access to the deep small intestine and, with the aid of the water jet function, facilitated precise submucosal dissection using WPM, thereby overcoming the technical difficulties of small intestinal ESD.

Endoscopy_UCTN_Code_TTT_1AP_2AD
